# Mating Interactions between *Schistosoma bovis* and *S. mansoni* and Compatibility of Their F1 Progeny with *Biomphalaria glabrata* and *Bulinus truncatus*

**DOI:** 10.3390/microorganisms10061251

**Published:** 2022-06-19

**Authors:** Amos Mathias Onyekwere, Alejandra De Elias-Escribano, Julien Kincaid-Smith, Sarah Dametto, Jean-François Allienne, Anne Rognon, Maria Dolores Bargues, Jérôme Boissier

**Affiliations:** 1 Department of Biology, Alex Ekwueme Federal University, Ndufu-Alike, Abakaliki PMB 1010, Nigeria; onyekwereamos@gmail.com; 2 IHPE, University Montpellier, CNRS, Ifremer, University Perpignan Via Domitia, 66000 Perpignan, France; kincaid-smith.julien@ird.fr (J.K.-S.); sarah.dametto@univ-perp.fr (S.D.); allienne@univ-perp.fr (J.-F.A.); rognon@univ-perp.fr (A.R.); 3 Departamento de Parasitología, Facultad de Farmacia, Universidad de Valencia, Av. Vicent Andrés Estellés s/n, 46100 Burjassot, Valencia, Spain; alejandra.elias@uv.es (A.D.E.-E.); m.d.bargues@uv.es (M.D.B.); 4 CIBER de Enfermedades Infecciosas, Instituto de Salud Carlos IIII, C/Monforte de Lemos 3-5, Pabellón 11, Planta 0, 28029 Madrid, Spain; 5 CBGP, IRD, CIRAD, INRAE, Institut Agro, University Montpellier, 34980 Montpellier, France

**Keywords:** *Schistosoma bovis*, *Schistosoma mansoni*, mating interactions, F1 progeny, compatibility, *Bulinus truncatus*, *Biomphalaria glabrata*

## Abstract

Contrary to the majority of other Trematoda, *Schistosoma* species are gonochoric. Consequently, in endemic areas where several schistosome species overlap and can co-infect the same definitive host, there may be frequent opportunities for interspecific pairing. Our experimental study provides novel insight on the pairing behavior between *Schistosoma bovis* and *S. mansoni* in mixed infections in mice. We used six mate choice experiments to assess mating interactions between the two schistosome species. We show that mating between the two *Schistosoma* species is not random and that *S. mansoni* exhibits greater mate recognition compared to *S. bovis*. We also performed reciprocal crosses (male *S. mansoni* × female *S. bovis*) and (female *S. mansoni* × male *S. bovis*) that produce active swimming miracidia. These miracidia were genotyped by ITS2 sequencing and proposed for mollusc infection. Molecular analyses show that all the miracidia are parthenogenetically produced (i.e., their harbor the mother ITS2 genotype) and as a consequence can only infect the mollusc of the maternal species. Offspring produced by male *S. mansoni* × female *S. bovis* pairing can only infect *Bulinus truncatus* whereas offspring produced by female *S. mansoni* × male *S. bovis* can only infect *Biomphalaria glabrata* snails. Evolutionary and epidemiological consequences are discussed.

## 1. Introduction

The class Trematoda is a diversified phylum of worms characterized by their parasitic way of life. Trematoda are usually hermaphroditic; however, the hundred species belonging to the family of Schistosomatidae are exceptional because they are gonochoric [1,2]. This family contains some species of considerable medical and veterinary importance [3,4]. Among them, the *Schistosoma* genus is the most important in terms of human health and social-economic impacts. Human schistosomiasis affects about 250 million people in about 78 countries worldwide, with the largest disease burden throughout sub-Saharan Africa [5]. The *Schistosoma* genus is composed of about 23 recognized species with at least 19 species that infect livestock and wild animals [6]. Although only five species are of veterinary importance to domestic animals, at least six species infect humans and are of medical interest [6]. Because several of these species are co-endemic and can share the same definitive host, interspecific crossing opportunities may occur. Several studies, both in the field and in the lab have addressed the potentiality of different *Schistosoma* species to encounter, to mate and to interbreed.

In the field, numerous studies have reported the existence of several potential interspecific crosses between different species of schistosomes [7]. Early reports were mainly based on the physical appearance of the eggs [8]. However, the viability of these eggs was not evaluated and these early physical observations have often been considered as misdiagnosis [7]. The use of molecular techniques has later confirmed hetero-specific pairing or resulting hybrid progeny between different schistosome species, when adult worms or miracidia were genotyped, respectively [7]. Natural interspecific hybridization may occur between human *Schistosoma* species (*S. haematobium* × *S. guineensis* or *S. mansoni*), animal schistosomes (*S. bovis* × *S. curasonni*) or between human and animal schistosomes (*S. haematobium* × *S. bovis* or *mattheei* or *curasonni* or *S. mansoni* × *S. rodhaini*). Interspecific pairing may induce some important outcomes for the parasites’ transmission in the field. For example, in Cameroon, hybridization between *S. haematobium* and *S. guineensis* has led to the local extinction of the latter species and the establishments of the former and their hybrid offspring [9,10]. Another major outcome is the zoonotic potential of some crosses as it has been observed in *S. haematobium* × *S. bovis* crosses [11,12].

In parallel to field identification, experimental approaches allowed to analyze interspecific interactions and to infer some biological aspects, such as mate choice, mate competition or the genetic outcome of the crosses, that cannot be address as easily in the field. Several interbreeding experiments in the laboratory have confirmed that *Schistosoma* species can successfully hybridize for several generations [13]. These experimental studies have evidenced either random mating or a preponderance of homo-specific pairing according to the phylogenetic distance of the interacting species [13]. When closely related species interact such as *S. haematobium* × *S. bovis* [14], *S. bovis* × *S. curassoni* [15], *S. haematobium* × *S. intercalatum* [16] or *S. intercalatum* × *S. guineensis* [17] the pairing is random. All these latter species are included in the same monophyletic *S. haematobium* group [3]. At the opposite when the species belong to two different evolutionary lineages mate preference is observed as evidenced for *S. mansoni* × *S. intercalatum* crosses [18]. The genetic background of the resulting progeny may also depend on the phylogenetic distance between the interacting species, ranging from parthenogenetic individuals [19] to substantial genomic introgression [14,20,21]. One visible consequence of such genetic signatures may be observed at the parasites’ life history trait level, and in particular for the parasite-mollusc compatibility. When the progeny is partheno-genetically produced, the mollusc host spectrum is limited to the host spectrum of the maternal schistosome species [19]. On the contrary,, for closely related species, hybridization can enlarge the host spectrum as evidenced in *S. haematobium* × *S. intercalatum* crosses [22].

The present study addresses inter-specific interactions between *S. mansoni* and *S. bovis* in the laboratory. These species belong to two different evolutionary lineages of schistosomes; the *S. mansoni* and the *S. haematobium* groups, respectively. *S. mansoni* is known to infect Humans, Non-Human Primates or rodents, while *S. bovis* is known to infect livestock and rodents [6]. In the field, both parasite species can share the same rodent host, as has been found in *Mastomys huberti* and *Arvicanthis niloticus* in Senegal [23]. This last study also evidenced interspecific pairing between an *S. mansoni* male and a *S. haematobium* × *S. bovis* hybrid female in *Mastomys huberti* [23]. A single study has experimentally exposed rodents to mixed infections between male *S. bovis* and female *S. mansoni* [24]. This last author has observed eggs with typical maternal species shape, but few contained viable miracidia and the work failed to infect *Bulinus* snail. In the current context of schistosomes’ potential zoonotic transmission, this study proposes to experimentally analyze *S. mansoni* and *S. bovis* interactions through: (i) mate interactions thanks to mate choice experiments; (ii) the compatibility between the progeny and the parental mollusc species (*Biomphalara glabrata* and *Bulinus truncatus*) after forced reciprocal crosses; and (iii) the nuclear genetic background of the progeny.

## 2. Materials and Methods

### 2.1. The Ethics Statement

This research was carried out according to national ethical standards established in the write of 1st February 2013 (NOR: AGRG1238753A), setting the conditions for approval, planning and operation of establishments, breeders and suppliers of animals used for scientific purposes and controls. The experiments carried out for this study were approved and provided a permit A66040 for animal experimentation by the French Ministry of Agriculture and Fishery (Ministere de l’Agriculture et de la Peche), and the French Ministry for Higher Education, Research and Technology (Ministere de l’Education Nationale de la Recherché et de la Technologie). The investigator has the official certificate for animal experimentation, obtained from both ministries (Decret n° 87/848 du 19 octobre 1987; number of authorization 007083).

### 2.2. Origin and Maintenance of Schistosome Strains

*Schistosoma bovis* and *S. mansoni* were maintained in the laboratory using *Bulinus truncatus* (Spanish strain) and *Biomphalaria glabrata* (Brazilian strain), respectively. The definitive hosts used were Swiss OF1 mice (Charles River Laboratories L’abresle, Saint-Germain-Nuelles, France). The parasite strains *S. bovis* and *S. mansoni* originated from Yegua-Salamanca (Spain) and Recife (Brazil), respectively. The *S. bovis* strain isolated in the early 1980s originates from Villar de la Yegua-Salamanca, and was provided by Ana Oleaga from the Spanish laboratory of parasitology of the Institute of Natural Resources and Agrobiology in Salamanca [25]. The *S. mansoni* strain isolated in 1975 originates from Recife (Brazil) and was provide by Pr. Y. Golvan from the Faculty of Medicine St Antoine, Paris (France). Compatibility of the F1 progeny was tested on four mollusc strains: two *B. truncatus* (from Spain and from Morocco) and two *B. glabrata* (from Brazil and from Guadeloupe).

### 2.3. Experimental Protocol

#### 2.3.1. Snail Infection and Obtention of Unisexual Clonal Population of Schistosome

Methods for miracidium recovery follow the previously published procedure [26,27]. Seventy-two *Biomphalaria glabrata* snails were exposed individually overnight to a single miracidium of *S. mansoni* each and 96 *Bulinus truncatus* snails were individually exposed overnight to a single miracidium of *S. bovis* each so that each infected snail would produce single-sex cercarial population. The molluscs were separated into two breeding tanks according to species and fed *ad libitum* for a duration of 35 days for miracidium to develop to cercariae. Molluscs placed in 24 well-plates according to species were stimulated under light for cercariae shedding. After 2–3 h, the 24 well-plates were examined under a binocular microscope for the presence of cercariae and any snail found to emit cercariae was assigned with an identity number. Three cercariae from each infected snail were individually captured for molecular sexing and each snail was separated into a plastic cup and fed *ad libitum*. Molecular sexing of *S. mansoni* and *S. bovis* cercariae was performed according to [14,28] respectively (see Supplementary Materials for detailed molecular biology protocols [28,29]). The snails were finally separated into four distinct tanks according to sex and species of cercariae.

#### 2.3.2. Mice Infection, Parasite Recovery and Species Identification

Mice were infected using the paddling method and worms were recovered thanks to hepatic perfusion technique. Details for mice infection and parasite recovery follow previously published procedure [26,27]. The sex, species and number of cercariae combination used for each mouse exposure are shown in Table 1.

The experimental design to quantify the frequency of homo- and hetero-specific coupling between *S. bovis* and *S. mansoni* is composed of five experiments (n°1–5—Table 1). Experiments n°1–4 aimed to test the individual choice of each species and sex. In experiments n°1 and n°2, we tested the female choice for *S. mansoni* and *S. bovis*. In experiments n°3 and n°4, we tested the male choice for *S. mansoni* and *S. bovis*. Experiments n°1–4 served as a restricted choice of mate where excess of one sex of the two species competing for pairing will ensure that all individuals of the other sex (that had the choice for homo- or hetero-specific mating) will be paired. Experiment n°5 served as full choice of mate. Mice were infected with the same number of cercariae of both sexes and species so that we could evaluate all paring combinations simultaneously. Experiment n°6 consists in producing F1 and F1’ progeny through forced reciprocal mating experiments. These last crosses were designed to obtain a first generation of miracidia to know whether these progenies are compatible with the snail intermediate host.

After cercarial exposure, the mice were euthanized at two months and adult worms recovered by hepatic perfusion. We used a magnifier lens and a small paintbrush to separate the dimorphic worms according to their sex (male or female). Each worm, whether mated or unmated, was placed in a 1.5 micro tube and labeled appropriately. Tubes containing worms were stored in the freezer at −20 °C for genetic analysis. The species of all worms (mated and unmated) where identified after DNA extraction using amplification methods (see Supplementary Materials for detailed molecular biology protocols).

#### 2.3.3. Mollusc Exposition with F1 and F1’ Miracidia

Albino mice exposed to male *S. mansoni* × female *S. bovis* cercariae and vice versa (F1 & F1’, see Experiments n°6, Table 1), were euthanized at two months post-cercarial exposure and eggs from the livers were hatched to recover first generation miracidia [26,27]. Forty miracidia of each cross were stored on Whatman FTA cards [30]. The sex of these miracidia was determined by PCR and a part of the ITS2 gene was sequenced for 66 specimens (see molecular biology methods Supplementary Materials for details). Sequences obtained were compared to reference sequences from Genbank database (AF531314.1 for *S. mansoni* and FJ588862.1 for *S. bovis*). We used 24 well-plates to expose 48 *Biomphalaria glabrata* snails each of Brazil and Guadeloupe strains individually overnight with 10 F1 or F1’ miracidia. The same procedure was used to expose 48 *Bulinus truncatus* (Morocco and Spain strains). The protocol for mollusc infection with F1 and F1’ miracidia is shown in Table 2 below. The molluscs were separated into eight breeding tanks according to their infected miracidia (F1 or F1’), snail species (*B. glabrata* or *B. truncatus*) and snail strain (Brazil, Guadeloupe, Morocco, or Spain). Snails were fed ad libitum for a duration of 60 days for miracidia to develop into cercariae. Molluscs placed in 24 well-plates according to group were stimulated under light to emit cercariae.

### 2.4. Statistical Analysis

The total number of adult worms recovered for each schistosome species was counted, e.g., homo-specific pairs, hetero-specific pairs, and single worms. We used the null hypothesis of random pairing to calculate the expected number of single and paired worms, e.g., in experiment 1 the expected number of homo-specific paired *S. bovis* females equals the total number of *S. bovis* females, times the total number of *S. bovis* males over the total number of males. We used the Chi-square tests with Yates correction for continuity to compare the expected and observed numbers of homo- and hetero-specific pairs. The p-value was adjusted for multiple comparisons using the Benjamini & Hochberg method. Statistical analysis was done using R Studio v1.4.1106.

## 3. Results

For each experiment, Table 3 shows the sex and species of the choosing partner. Random pairing is observed when *S. bovis* is the species that can choose whether male (x^2^ = 2.73; *p* = 0.8 after Benjamini & Hochberg correction) or female (x^2^ = 0.29; *p* = 1.00 after Benjamini & Hochberg correction). Homo-specific pairs are more numerous than heterospecific pairs when *S. mansoni* is the species that can choose whether male (x^2^ = 9.85; *p* = 0.016 after Benjamini & Hochberg correction) or female (x^2^ = 7.66; *p* = 0.048 after Benjamini & Hochberg correction). Table 4 shows the number of homo-, hetero-specific pairs and single worms when mice are exposed to equal number of cercariae whatever their sex and their species. The number of homo-specific pairs is bigger than expected under the hypothesis of random association (x^2^ = 31.86; *p* < 0.001 after Benjamini & Hochberg correction). However, no ♂Sb × ♀Sm pairs were observed because all *S. mansoni* female were monopolized by male *S. mansoni.*

Table 5 shows compatibility of F1 (female *S. bovis* x male *S. mansoni*) and F1’ (male *S. bovis* × female *S. mansoni*) miracidia with the intermediate snail hosts of both schistosomes’ parental species. F1’ miracidia readily infect *B. glabrata* from Brazil (50%) and from Guadeloupe (20.8%). F1 miracidia readily infect in *B. truncatus* from Spain (10.6%) and from Morocco (3.3%). Loss of compatibility was noted in F1 miracidia for *Biomphalaria* and in F1’ miracidia for *Bulinus* snail. Both sexes were identified in the F1 and F1’ progenies. Among the F1 miracidia, 9 and 24 were female and male, respectively. Among the F1’ miracidia, 15 and 18 were female and male, respectively. 505 base pairs of the ITS2 nuclear gene have been sequenced for 66 miracidia. On the 33 sequences of the F1 miracidia all exhibit a *S. bovis* gene profile (no heterozygous profile) and on the 33 sequences of the F1’ miracidia all exhibit a *S. mansoni* gene profile (no heterozygous profile).

## 4. Discussion

Several studies have shown that, in experimental mixed infections, there are no physiological barriers preventing encounters and mating of schistosomes of different species, even species belonging to a different genus in the definitive host [19]. Our overall findings on the experimental combinations between *S. mansoni* males’ × *S. bovis* females and vice versa demonstrated that (i) mating between the two *Schistosoma* species is not random; (ii) *S. mansoni* exhibits greater mate recognition compared to *S. bovis;* (iii) the progeny is parthenogenetic; and, as a consequence, (iv) the mollusc host spectrum of the F1 progeny is limited to the maternal schistosome species host spectrum.

Random pairing has been observed in crosses between *S. haematobium* × *S. bovis* [31], *S. bovis* × *S. curasonni* [15], *S. haematobium* × *S. intercalatum* [16] and *S. intercalatum* × *S. guineensis* [17]. Our study shows mate choice recognition, and is in tandem with results obtained in mixed infections of *S. haematobium* × *S. mattheei* [32] or *S. mansoni* × *S. intercalatum* [18]. Mate recognition seems to be dependent on the genetic proximity of the interacting species: *S. bovis, S. curassoni, S. intercalatum, S. haematobium* and *S. guineensis* are more related among each other compared to *S. haematobium* and *S. mattheei,* and even less so between *S. mansoni* and *S. bovis* or *S. intercalatum*.

Our study also shows that *S. mansoni* exhibited greater specific mate preference than *S. bovis* and this indicates that intra-*S. mansoni* recognition is stronger than intra-*S. bovis*; evidence of *S. mansoni* exhibiting greater mate recognition than *S. intercalatum* [18] and *S. haematobium* exhibiting greater mate recognition than *S. mattheei* [32] has been reported and our results further buttress the existence of mechanisms in *Schistosoma* species favoring the pairing of homo-specific partners. In addition, it has been reported that, whatever their genotype, *S. mansoni* males show a stronger competitiveness at coupling with females than *S. intercalatum* males [33,34]. It has been demonstrated that the former will change partner to mate with conspecific females in preference to hetero-specific females whenever the opportunity arises [33,34]. It has also been evidenced that, in the absence of *S. mansoni* female worms, unpaired *S. mansoni* male worms that arrive in a pre-established *S. intercalatum* infection are more competitive and can pull away female *S. intercalatum* from male *S. intercalatum* [33,34].

Depending on the evolutionary lineage of the species, the interspecies sexual interactions in schistosomes may lead to either hybrids or parthenogenetic offspring [19,24]. Our experimental design on mating interactions between *S. mansoni* males’ × *S. bovis* females (two species of schistosomes that belong to two different evolutionary lineages) and vice versa shows that the F1 progenies are parthenogenetically produced. Within the family of schistosomatidae, parthenogenesis has been reported in some species [13,19]. Apart from female *Schistosomatium douthitti,* which regularly mature and produce numerous viable eggs in unisexual infections [35], the induction of parthenogenesis in other female schistosomes has been evidenced in crosses with males from a different species group. Induced parthenogenesis has been observed in female *S. mansoni* stimulated by either *S. japonicum*, *S. intercalatum* or *S. douthitti* male, and in female *S. japonicum* or female *S. mattheei* stimulated by male *S. mansoni* [19]. Cytogenetic studies on the progeny of these last crosses have shown that all type of parthenogenesis can be observed: haploid, apomictic diploid or automictic diploic; the haploid parthenogenesis seems to be the most frequent mechanism [19]. Because the parthenogenetic status of the *S. mansoni* × *S. bovis* F1 progeny was assessed by sequencing a nuclear gene, we cannot infer the ploidy of this progeny. Finally, because F1 progeny only harbor the mother genotype, it can only infect the snail corresponding to the mother species (*B. truncatus* for *S. bovis* and *B. glabrata* for *S. mansoni*). A similar result has been observed in *S. japonicum* × *S. mansoni* crosses, where female *S. japonicum* × male *S. mansoni* can only infect *Oncomelania hupensis* snail while where female *S. mansoni* × male *S. japonicum* can only infect *B. glabrata* [36].

## 5. Conclusions

The production of hybrid offspring in laboratory experiments is a useful approach to determine levels of zoonotic potential in schistosome species. This can also help predict if hybrid offspring could be evidenced in the field. Our study demonstrated that pairing is possible between *S. mansoni* and *S. bovis* with the production of viable parthenogenetic offspring, but with a limitation in their ability to infect both parental mollusc hosts. Theoretically, F1 *S. mansoni* × *S. bovis* miracidia could be evidenced in the field at least in rodent host, where both parasites’ species can be found [23]. The barrier between schistosomes infecting human or animal has recently been challenged with the discovery of widespread *S. haematobium* × *S. bovis* hybrids in several West African countries [11,12,37,38,39]. Even if *S. mansoni* are not closely related to *S. bovis,* contrary to *S. haematobium* they have the same tropism for the mesenteric vein system and *S. mansoni* × *S. bovis* could therefore also be evidenced in human feces. Without molecular biology, a female *S. mansoni* × male *S. bovis* F1 progeny not be identified because it harbors the expected *S. mansoni* egg shape and infects the host attributed to *S. mansoni* transmission. However, *S. bovis* egg shape in human feces might attract attention. The presence of *S. bovis* egg shape in human feces has already been evidenced but attributed to contamination after the ingestion of cows infected by *S. bovis* parasite. However, this type of observation is very rare and the fact that the F1 individuals produced are parthenogenetic should strongly limit the spread of this hybrid. Interestingly our results may also have implications for schistosomiasis transmission and thus its control. They suggest that co-infection and the formation of heterospecific pairs in reservoir hosts such as rodents, although not leading to hybridization, is sufficient to allow female of each species to perpetuate the parasites life cycles even in the absence of conspecific mates. This supports the fact that focusing on reducing human schistosomiasis alone may not be sufficient for sustainable control, especially if animal-infecting species can stimulate the transmission of single-sex human-infecting species.

## Figures and Tables

**Table 1:** Number of cercariae used for each experiment according to sex and species of parasite.

Experiment Number	S. *bovis*		*S. mansoni*		Number of Mice
	Males	Females	Males	Females	
Limited choice experiments					
1. limiting sex: female *S. bovis*	60	60	60	--	8
2. limiting sex: female *S. mansoni*	60	--	60	60	8
3. limiting sex: male *S. bovis*	60	60	--	60	8
4. limiting sex: male *S. mansoni*	--	60	60	60	8
Full choice experiment					
5. no limiting sex	45	45	45	45	8
Forced reciprocal mating experiment					
6. F1 progeny	--	100	100	--	4
6. F1’ progeny	100	--	--	100	4

**Table 2:** Snail infection with F1/F1’ miracidia for compatibility testing. Sb: *S. bovis*, Sm: *S. mansoni*.

Exp	Intermediate Snail Host (Strain)	Number of Snails Exposed	Progeny
A	*Biomphalaria glabrata* (Brazil)	48	♂Sb × ♀Sm (F1’)
B	*Biomphalaria glabrata* (Guadeloupe)	48	♂Sb × ♀Sm (F1’)
C	*Biomphalaria glabrata* (Brazil)	48	♀Sb × ♂Sm (F1)
D	*Biomphalaria glabrata* (Guadeloupe)	48	♀Sb × ♂Sm (F1)
E	*Bulinus truncatus* (Morocco)	48	♂Sb × ♀Sm (F1’)
F	*Bulinus truncatus* (Spain)	48	♂Sb × ♀Sm (F1’)
G	*Bulinus trancatus* (Morocco)	48	♀Sb × ♂Sm (F1)
H	*Bulinus trancatus* (Spain)	48	♀Sb × ♂Sm (F1)

**Table 3:** Summarized information (limited choice experiments: exp. 1–4), to show numbers of paired (homo- and hetero-specific) and unpaired worms recovered from mice exposed to a limited combination of cercariae. Expected numbers of pairs under random mating is shown in brackets. Sb: *S. bovis*, Sm: *S. mansoni*.

Exp	Host	Choosing Partner	Homo-Specific Pairs	Hetero-Specific Pairs	Single Worms	
1.	Mouse	♀Sb	♀Sb × ♂Sb	♀Sb × ♂Sm	♂Sm	♂Sb
	1		2	0	5	4
	2		8	4	7	4
	3		1	0	5	6
	4		5	3	5	3
	5		6	6	5	5
	6		4	3	4	5
	7		1	12	0	1
	8		1	1	1	0
Total			28 (30)	29 (27)	32	24
2.		♀Sm	♀Sm × ♂Sm	♀Sm × ♂Sb	♂Sm	♂Sb
	1		5	1	7	8
	2		13	4	0	1
	3		8	1	6	4
	4		12	0	6	13
	5		4	3	0	0
	6		8	2	0	3
	7		9	1	0	1
	8		7	6	0	0
Total			66 (54)	18 (30)	19	30
3.		♂Sb	♂Sb × ♀Sb	♂Sb × ♀Sm	♀Sm	♀Sb
	1		4	2	2	3
	2		1	3	6	4
	3		1	7	2	2
	4		3	6	4	8
	5		0	5	4	4
	6		0	3	2	1
	7		0	1	7	4
	8		2	2	2	4
Total			11 (16)	29 (24)	29	30
4.		♂Sm	♂Sm × ♀Sm	♂Sm × ♀Sb	♀Sm	♀Sb
	1		15	1	11	0
	2		8	0	1	4
	3		4	0	5	4
	4		5	0	0	0
	5		9	1	0	2
	6		8	2	0	9
	7		6	2	0	1
	8		12	3	0	3
Total			67 (55)	9 (21)	17	23

**Table 4:** Summarized information of homo-specific pairs, hetero-specific pairs and unpaired worms recovered from mice exposed to simultaneous infections to full combinations of cercariae. Expected number of pairs under random mating is shown in brackets. Sb: *S. bovis*, Sm: *S. mansoni*.

Exp 5.	Homo-Specific	Homo-Specific	Hetero-Specific	Hetero-Specific	Single Worms			
Mouse	♂Sm × ♀Sm	♂Sb × ♀Sb	♂Sm × ♀Sb	♂Sb × ♀Sm	♂Sm	♂Sb	♀Sm	♀Sb
1	6	3	1	0	3	4	0	0
2	4	2	3	0	1	1	0	0
3	5	3	6	0	3	3	0	1
4	5	5	3	0	0	1	0	0
5	9	4	4	0	4	2	0	1
6	6	2	2	0	1	2	0	0
7	5	3	5	0	0	0	0	2
8	6	5	1	0	0	1	0	0
Total	46 (30)	27 (18)	25 (36)	0 (15)	12	14	0	4

**Table 5:** Compatibility of F1 progeny of *S. bovis* (male) × *S. mansoni* (female) and *S. mansoni* (male) × *S. bovis* (female) in *Biomphalaria glabrata* and *Bulinus truncatus*. Each snail was exposed to 12 miracidia. Sb: *S. bovis*, Sm: *S. mansoni*.

Exp	Snail Species	Progeny	Snails Exposed	Snails Surviving	Snails Infected	% of Snails Infected
A	*B. glabrata* (Brazil)	♂Sb × ♀Sm (F1’)	48	46	23	50
B	*B. glabrata* (Guadeloupe)	♂Sb × ♀Sm (F1’)	48	48	10	20.8
C	*B. glabrata* (Brazil)	♀Sb × ♂Sm (F1)	48	48	0	0
D	*B. glabrata* (Guadeloupe)	♀Sb × ♂Sm (F1)	48	48	0	0
E	*B. truncatus* (Morocco)	♂Sb × ♀Sm (F1’)	48	29	0	0
F	*B. truncatus* (Spain)	♂Sb × ♀Sm (F1’)	48	45	0	0
G	*B. truncatus* (Morocco)	♀Sb × ♂Sm ♂ (F1)	48	30	1	3.3
H	*B. truncatus* (Spain)	♀Sb × ♂Sm ♂ (F1)	48	47	5	10.6

## Supplementary Materials

The following supporting information can be downloaded at: https://www.mdpi.com/article/10.3390/microorganisms10061251/s1. Molecular Biology Protocols. References [40,41] cited in Supplementary Materials.

## References

[1] Loker E.S., Brant S.V. (2006). Diversification, dioecy and dimorphism in schistosomes. Trends Parasitol..

[2] Mone H., Boissier J. (2004). Sexual biology of schistosomes. Adv. Parasitol..

[3] Lockyer A.E., Olson P.D., Ostergaard P., Rollinson D., Johnston D.A., Attwood S.W., Southgate V.R., Horak P., Snyder S.D., Le T.H. (2003). The phylogeny of the Schistosomatidae based on three genes with emphasis on the interrelationships of *Schistosoma* Weinland, 1858. Parasitology.

[4] Boissier J., Mouahid G., Moné H. (2019). Schistosoma spp..

[5] Lo N.C., Bezerra F.S.M., Colley D.G., Fleming F.M., Homeida M., Kabatereine N., Kabole F.M., King C.H., Mafe M.A., Midzi N. (2022). Review of 2022 WHO guidelines on the control and elimination of schistosomiasis. Lancet Infect. Dis..

[6] Panzner U., Boissier J. (2021). Natural Intra- and Interclade Human Hybrid Schistosomes in Africa with Considerations on Prevention through Vaccination. Microorganisms.

[7] Leger E., Webster J.P. (2017). Hybridizations within the Genus *Schistosoma*: Implications for evolution, epidemiology and control. Parasitology.

[8] Alves W. (1948). Observations on *S. mattheei* and *S. haematobium*—Adults from experimental animals and man. Trans. R. Soc. Trop. Med. Hyg..

[9] Tchuem Tchuente L.A., Southgate V.R., Jourdane J., Webster B.L., Vercruysse J. (2003). *Schistosoma intercalatum*: An endangered species in Cameroon?. Trends Parasitol..

[10] Webster B.L., Tchuem Tchuente L.A., Jourdane J., Southgate V.R. (2005). The interaction of *Schistosoma haematobium* and *S. guineensis* in Cameroon. J. Helminthol..

[11] Savassi B., Dobigny G., Etougbetche J.R., Avocegan T.T., Quinsou F.T., Gauthier P., Ibikounle M., Mone H., Mouahid G. (2021). *Mastomys natalensis* (Smith, 1834) as a natural host for *Schistosoma haematobium* (Bilharz, 1852) Weinland, 1858 x *Schistosoma bovis* Sonsino, 1876 introgressive hybrids. Parasitol. Res..

[12] Savassi B., Mouahid G., Lasica C., Mahaman S.K., Garcia A., Courtin D., Allienne J.F., Ibikounle M., Mone H. (2020). Cattle as natural host for *Schistosoma haematobium* (Bilharz, 1852) Weinland, 1858 x *Schistosoma bovis* Sonsino, 1876 interactions, with new cercarial emergence and genetic patterns. Parasitol. Res..

[13] Southgate V.R., Jourdane J., Tchuem Tchuenté L.A. (1998). Recent studies on the reproductive biology of the schistosomes and their relevance to speciation in the digenea. Int. J. Parasitol..

[14] Kincaid-Smith J., Tracey A., de Carvalho Augusto R., Bulla I., Holroyd N., Rognon A., Rey O., Chaparro C., Oleaga A., Mas-Coma S. (2021). Morphological and genomic characterisation of the *Schistosoma* hybrid infecting humans in Europe reveals admixture between *Schistosoma haematobium* and *Schistosoma bovis*. PLoS Negl. Trop. Dis..

[15] Rollinson D., Southgate V.R., Vercruysse J., Moore P.J. (1990). Observations on natural and experimental interactions between *Schistosoma bovis* and *S. curassoni* from West Africa. Acta Trop..

[16] Southgate B.A., Rollinson D., Ross A.G., Knowles N.G. (1982). Mating behaviour in mixed infections of *Schistosoma haematobium* and *S. intercalatum*. J. Nat. Hist..

[17] Pages J.R., Southgate V.R., Tchuem Tchuente L.A., Jourdane J. (2001). Lack of prezygotic isolation by assortative mating between the two cryptic species of the polytypic *Schistosoma intercalatum* taxon. Parasitol. Res..

[18] Tchuem Tchuenté L.A., Imbert-Establet D., Delay B., Jourdane J. (1993). Choice of mate, a reproductive isolating mechanism between *Schistosoma intercalatum* and *S. mansoni* in mixed reflexions. Int. J. Parasitol..

[19] Jourdane J., Imbert-Establet D., Tchuente L.A. (1995). Parthenogenesis in schistosomatidae. Parasitol. Today.

[20] Platt R.N., McDew-White M., Le Clec’h W., Chevalier F.D., Allan F., Emery A.M., Garba A., Hamidou A.A., Ame S.M., Webster J.P. (2019). Ancient Hybridization and Adaptive Introgression of an Invadolysin Gene in Schistosome Parasites. Mol. Biol. Evol..

[21] Rey O., Toulza E., Chaparro C., Allienne J.F., Kincaid-Smith J., Mathieu-Begne E., Allan F., Rollinson D., Webster B.L., Boissier J. (2021). Diverging patterns of introgression from *Schistosoma bovis* across *S. haematobium* African lineages. PLoS Pathog..

[22] Webster B.L., Southgate V.R. (2003). Compatibility of *Schistosoma haematobium*, *S. intercalatum* and their hybrids with *Bulinus truncatus* and *B. forskalii*. Parasitology.

[23] Catalano S., Sene M., Diouf N.D., Fall C.B., Borlase A., Leger E., Ba K., Webster J.P. (2018). Rodents as Natural Hosts of Zoonotic Schistosoma Species and Hybrids: An Epidemiological and Evolutionary Perspective from West Africa. J. Infect. Dis..

[24] Taylor M.G. (1970). Hybridisation experiments on five species of African schistosomes. J. Helminthol..

[25] Silva M.L., Vicente F.S., Avelino I.C., Martin V.R. (1977). Susceptibility of *Planorbarius metidjensis* from Portugal and Spain to *Schistosoma bovis* from Salamanca, Spain. Malacologia.

[26] Boissier J., Chlichlia K., Digon Y., Ruppel A., Mone H. (2003). Preliminary study on sex-related inflammatory reactions in mice infected with *Schistosoma mansoni*. Parasitol. Res..

[27] Boissier J., Mone H. (2000). Experimental observations on the sex ratio of adult *Schistosoma mansoni*, with comments on the natural male bias. Parasitology.

[28] Portela J., Grunau C., Cosseau C., Beltran S., Dantec C., Parrinello H., Boissier J. (2010). Whole-genome in-silico subtractive hybridization (WISH)--using massive sequencing for the identification of unique and repetitive sex-specific sequences: The example of *Schistosoma mansoni*. BMC Genom..

[29] Kincaid-Smith J., Boissier J., Allienne J.F., Oleaga A., Djuikwo-Teukeng F., Toulza E. (2016). A Genome Wide Comparison to Identify Markers to Differentiate the Sex of Larval Stages of *Schistosoma haematobium, Schistosoma bovis* and their Respective Hybrids. PLoS Negl. Trop. Dis..

[30] Kebede T., Bech N., Allienne J.F., Olivier R., Erko B., Boissier J. (2020). Genetic evidence for the role of non-human primates as reservoir hosts for human schistosomiasis. PLoS Negl. Trop. Dis..

[31] Kincaid-Smith J., Mathieu-Begne E., Chaparro C., Reguera-Gomez M., Mulero S., Allienne J.F., Toulza E., Boissier J. (2021). No pre-zygotic isolation mechanisms between *Schistosoma haematobium* and *Schistosoma bovis* parasites: From mating interactions to differential gene expression. PLoS Negl. Trop. Dis..

[32] Southgate V.R., Tchuem Tchuente L.A., Vercruysse J., Jourdane J. (1995). Mating behaviour in mixed infections of *Schistosoma haematobium* and *S. mattheei*. Parasitol. Res..

[33] Tchuem Tchuenté L.A., Southgate V.R., Imbert-Establet D., Jourdane J. (1995). Change of mate and mate competition between males of *Schistosoma intercalatum* and *S. mansoni*. Parasitology.

[34] Tchuem Tchuenté L.A., Southgate V.R., Jourdane J. (1995). Mating competition between *Schistosoma mansoni* and *S. intercalatum*. Trans. R. Soc. Trop. Med. Hyg..

[35] Short R.B. (1952). Uniparental Miracidia of Schistosomatium douthitti and their Progeny (Tremaoda, Schistosomatidae). Am. Midl. Nat..

[36] Imbert-Establet D., Xia M., Jourdane J. (1994). Parthenogenesis in the genus *Schistosoma*: Electrophoretic evidence for this reproduction system in *S. japonicum* and *S. mansoni*. Parasitol. Res..

[37] Huyse T., Webster B.L., Geldof S., Stothard J.R., Diaw O.T., Polman K., Rollinson D. (2009). Bidirectional introgressive hybridization between a cattle and human schistosome species. PLoS Pathog..

[38] Onyekwere A.M., Rey O., Allienne J.F., Nwanchor M.C., Alo M., Uwa C., Boissier J. (2022). Population Genetic Structure and Hybridization of *Schistosoma haematobium* in Nigeria. Pathogens.

[39] Angora E.K., Boissier J., Menan H., Rey O., Tuo K., Toure A.O., Coulibaly J.T., Meite A., Raso G., N’Goran E.K. (2019). Prevalence and Risk Factors for Schistosomiasis among Schoolchildren in two Settings of Cote d'Ivoire. Trop. Med. Infect. Dis..

[40] Beltran S., Galinier R., Allienne J.F., Boissier J. (2008). Cheap, rapid and efficient DNA extraction method to perform multilocus microsatellite genotyping on all *Schistosoma mansoni* stages. Mem. Inst. Oswaldo Cruz..

[41] Webster B.L., Rollinson D., Stothard J.R., Huyse T. (2010). Rapid diagnostic multiplex PCR (RD-PCR) to discriminate *Schistosoma haematobium* and *S. bovis*. J. Helminthol..

