# Supplementary material for: Mating Interactions between Schistosoma bovis and S. mansoni and Compatibility of Their F1 Progeny with Biomphalaria glabrata and Bulinus truncatus
Source: Microorganisms. 2022 Jun 19;10(6):1251. doi: 10.3390/microorganisms10061251 (PMC9227498; doi:10.3390/microorganisms10061251)
Supplement: Supplementary file 1 [file microorganisms-10-01251-s001.zip › microorganisms-1755666-supplementary.pdf]

## MOLECULAR BIOLOGY PROTOCOLS

### DNA extraction [1]

Genomic DNA from either cercariae or worms were individually extracted using the hot shot method. In this method, 20µL of NaOH (250 mM) was dispensed into each well or tube containing the specimen. This was incubated at room temperature for 20-60 minutes before heating at 99◦C for 3 minutes. 20µL of the "mix" (neutralizing solution) which consist of 10µL HCl (250 mM), 5µL of Tris-HCl (500 mM) and 5µL Triton X-100 (2%) was added and a second heating at 99◦C was performed. A 1/10 dilution of the DNA extract was prepared using ultra-pure water which served as the working solution. The working solution (DNA) was stored in the freezer at -20◦C for PCR amplification.

### Sexing clonal population of *Schistosoma mansoni* cercariae or miracidia [2]:

*Polymerase Chain Reaction (PCR) amplification*

The primer sequences for sexing *S. mansoni* larvae are SmWSPP_4_F/R (F: 5'-TGTTTCGAATTTCACACTTCA -3', R: 5'- CATTCACAGTTTGGCGAACA-3') targeting a W chromosome specific sequence and rhodo3_F/R for positive control (F: 5'-GGAATTGGTACCGTCAGC-3' R: 5'- AGGTTCACTTTAGTGTGGC-3'). The PCR was performed at a final volume of 10µL. This consists of 5µL 2X multiplex PCR master mix (Qiagen, Hilden, Germany), 1µL 10X *S. mansoni* primers and 4µL of working DNA extract.

*PCR Running Program*

The thermal cycling was performed in a PerkinElmer 9600 thermal cycler (PerkinElmer, Waltham, Massachusetts, USA) and the PCR conditions used were: Pre-denaturing at 95◦C for 15 minutes, 45 cycles of 30 seconds at 94◦C (denaturing), 90 seconds at 56◦C (annealing), and 60 seconds at 72◦C (extending). This was followed by final extending period of 30 minutes at 72◦C. The PCR product was stored in the refrigerator (4-6°C) until used for gel electrophoresis.

*Gel electrophoresis*

2% of agarose gel was prepared, and 6µL of Midori dye was added to the gel. 2µL of green loading dye was added to each well of the PCR products with a multi-channel pipette to make a final volume of 12µ. 8µL of the PCR product was loaded in each of the wells of the agarose gel using multi-channel Pipette. The PCR products in the gel were analyzed by electrophoresis at 100 V for 30 minutes. The result was read in trans-illuminator, and the picture in the gel was taken.

### Sexing clonal population of *Schistosoma bovis* cercariae or miracidia ([3]):

*PCR amplification*

The primer sequences for sexing *S. bovis* larvae are W*ShSb*3_F/R (F: 5'-GGTGGTCAGGCATTGATTCT -3', R: 5'- CATGTTTAGGCGCTTCAGGT -3') targeting a W chromosome specific sequence and GAPDH for positive control (F: 5'-CGACCATTGATGCAGCTAAA -3' R: 5'- TTCCAAAATCCCCTTCATTG -3'). The PCR was performed at a final volume of 10µL. This consist of 5µL 2X multiplex PCR master mix (Qiagen, Hilden, Germany), 1µL 10X *S. mansoni* primers and 4µL of working DNA extract.

*PCR Running Program*

The thermal cycling was performed in a PerkinElmer 9600 thermal cycler (PerkinElmer, Waltham, Massachusetts, USA) and the PCR conditions used were: Pre-denaturing at 95◦C for 15 minutes, 45 cycles of 30 seconds at 94◦C (denaturing), 90 seconds at 56◦C (annealing), and 60 seconds at 72◦C (extending). This was followed by final extending period of 30 minutes at 72◦C. The PCR product was stored in the refrigerator (4-6◦C) until used for gel electrophoresis.

*Gel electrophoresis*

2% of agarose gel was prepared, and 6µL of Midori dye was added to the gel. 2µL of green loading dye was added to each well of the PCR products with a multi-channel pipette to make a final volume of 12µ. 8µL of the PCR product was loaded in each of the wells of the agarose gel using multi-channel Pipette. The PCR products in the gel was analyzed by electrophoresis at 100 V for 30 minutes. The result was read in trans-illuminator, and the picture in the gel was taken.

**Species identification by Rapid-Diagnostic-PCR**

*PCR amplification*

The species of each adult worm was identified using the Rapid Diagnostic pPlymerase Chain Reaction (RD-PCR) for mitochondrial DNA gene (Cox1 mtDNA) according to [4]. The primers we used were a single universal reverse primer; (Shmb.R, 5-CAA GTA TCA TGA AAY ART ATR TCT AA-3') and three species-specific forward primers; (Sh.F, 5'-GGT CTC GTG TAT GAG ATC CTA TAG TTT G-3') for *S. haematobium*, (Sm.F, 5'-CTT TGA TTC GTT AAC TGG AGT G-3') for *S. mansoni* and (Sb.F, 5'-GTT TAG GTA GTG TAG TTT GGG CTC AC-3') for *S. bovis*. Each PCR is made up of 1.2µL of ultra-pure water, 2µL of buffer (Green GoTaq Flexi buffer, 5X; Promega; Madison, Wisconsin, USA), 1.2µL of 25 mM MgCl2 (Promega), 0.4µL of 10 mM dNTPs mix (Promega), 1µL of 10X primer mix (4µL of 100µM reverse primer, 4µL of each 100µM forward primer and 84µL of ultra-pure water), 0.2µL of 5U/µL of GoTaq G2 Hot Start Polymerase (Promega), and 4µL of DNA extract, making a total volume of 10µL for the PCR mix.

*PCR Running Program*

Thermal cycling was performed in (plate thermal cycler) a PerkinElmer 9600 Thermal Cycler (PerkinElmer, Waltham, Massachusetts, USA) and the PCR conditions used were: pre-denaturing at 95°C for 3 minutes; 45 cycles of 10 seconds at 95°C (denaturing), 30 seconds at 52°C (annealing), and 10 seconds at 72°C (extending). This was followed by a final extending period of 2 minutes at 72°C. The PCR product was stored in the refrigerator at 4°C until used.

*Gel electrophoresis*

The PCR products (Cox1) was visualized on 2% agarose gel stained with 8µl Midori dye. 9µL of the PCR product was loaded into each well using multi-channel micro-pipette (including wells for positive controls: *S. mansoni* or *S. bovis* and water for negative control) and 4µL for size standard 100 bp (base-pair) ladder. The PCR products in the gel was analyzed by electrophoresis at 135 V for 30-35 minutes and transferred to the UV trans-illuminator where the picture in the gel was taken. Bands 215 bp or 260 bp revealed, *S. mansoni* or *S. bovis* respectively [4].

**Genetic signature of F1 hybrid offspring**

*PCR amplification*

The genetic signature of the hybrid miracidia was inferred by sequencing a part of the Internal Transcribed Spacer 2 (ITS2) nuclear gene. The ITS2 mix was performed with a single forward primer ITS_S2F: 5'-GGCTGCAGCGTTAACCATTA-3', and a single reverse primer ITS_S2R: 5'-ACACACACCATCGGTACAAA-3'. The PCR was performed at a final volume of 25µL. This consist of 12.8µ ultra-pure water, 5µL of green buffer, 1.5µL MgCL2, 0.5µL dNTPs, 1µL of X10 each primer mix, 0.2µL Gotaq Hot Start Polymerase and 3µL of working DNA extract.

*PCR running program*

The thermal cycling was performed in a PerkinElmer 9600 thermal cycler (PerkinElmer, Waltham, Massachusetts, USA) and the PCR conditions used were: Pre-denaturing at 95◦C for 3 minutes, 45 cycles of 40 seconds at 95◦ (denaturing), 40 seconds at 58◦C (annealing), and 40 seconds at 72C (extending). This was followed by final extending period of 5 minutes at 72◦C. The PCR product was stored in the refrigerator (4◦C) until used for gel electrophoresis.

*Gel electrophoresis and sequencing*

2% agarose gel was prepared and stained with 6µL Midori green dye. 4µL of PCR products was loaded in each gel well with multi-channel pipette and 4µL of size standard 100 bp ladder. Positive control of *S. bovis* was included. The PCR products in the gel was analyzed by electrophoresis at 135 V for 30 minutes. The result was read in the UV trans-illuminator, and the picture in the gel was taken. The expected fragment length was 505 bp. Sixty-six (66) samples were selected based on the quality of the amplicons. These successfully amplified PCR products were purified and sequenced on an Applied Biosystem Genetic Analyzer at Genoscreen, Lille, France.

1. Beltran, S.; Galinier, R.; Allienne, J.F.; Boissier, J. Cheap, rapid and efficient DNA extraction method to perform multilocus microsatellite genotyping on all *Schistosoma mansoni* stages. *Mem Inst Oswaldo Cruz* **2008**, *103*, 501-503.
2. Portela, J.; Grunau, C.; Cosseau, C.; Beltran, S.; Dantec, C.; Parrinello, H.; Boissier, J. Whole-genome in-silico subtractive hybridization (WISH)--using massive sequencing for the identification of unique and repetitive sex-specific sequences: the example of Schistosoma mansoni. *BMC Genomics* **2010**, *11*, 387.
3 Kincaid-Smith, J.; Boissier, J.; Allienne, J.F.; Oleaga, A.; Djuikwo-Teukeng, F.; Toulza, E. A Genome Wide Comparison to Identify Markers to Differentiate the Sex of Larval Stages of *Schistosoma haematobium, Schistosoma bovis* and their Respective Hybrids. *PLoS Negl. Trop. Dis.* **2016**, *10*, e0005138.
4. Webster, B.L.; Rollinson, D.; Stothard, J.R.; Huyse, T. Rapid diagnostic multiplex PCR (RD-PCR) to discriminate Schistosoma haematobium and S. bovis. *J Helminthol* **2010**, *84*, 107-114.
